# *In vivo* and *in vitro* Approach to Anti-arthritic and Anti-inflammatory Effect of Crocetin by Alteration of Nuclear Factor-E2-Related Factor 2/hem Oxygenase (HO)-1 and NF-κB Expression

**DOI:** 10.3389/fphar.2018.01341

**Published:** 2018-12-12

**Authors:** Yi Li, Rajat Kakkar, Jian Wang

**Affiliations:** ^1^Department of Joint Surgery, Shandong Provincial Hospital Affiliated to Shandong University, Jinan, China; ^2^Chandrasheker College of Pharmacy, Allahabad, India

**Keywords:** crocetin, enzymatic hydrolysis, arthritis, RAW 264.7 macrophage, inflammation

## Abstract

Crocetin (apo carotenoid dicarboxylic acid) is a common constituent of saffron. Its importance is well documented in Chinese medicine. Some studies have reported the inhibitory effect on inflammation in rats. The aim of the current experimental investigation to scrutinize the anti-inflammatory effect of Crocetin using the lipo polysaccharide (LPS) induced mouse macrophages (RAW 264.7) *in vitro* and complete Freund’s adjuvant-induced arthritis model and to explore *in vivo* possible mechanism of action. RAW 264.7 macrophages were used for estimation of the effect of crocetin on the cyclooxygenase (COX-2), nitric oxide (NO)production, anti-inflammatory and along with pro-inflammatory cytokines such as tumor necrosis factor-α (TNF-α), interleukin-1β (IL-1β), interleukin-6 (IL-6), and interleukin-10 (IL-10). Single intraperitoneal injection of complete freund’s adjuvant (CFA) was used to induce arthritis. The rats were divided into different group and received the oral administration of crocetin in a dose-dependent manner with indomethacin till 28 days. The paw edema and body weight was estimated at regular interval of time. The biochemical parameters, hematological and pro-inflammatory cytokines such as tumor necrosis factor receptor 1 (TNF-R1), IL-6, and IL-1β, Vascular endothelial growth factor (VEGF); heme oxygenase-1/nuclear factor erythroid 2–related factor 2 (HO-1/Nrf-2) expression were estimated at end of the experimental study. Crocetin inhibited the COX-2 catalyzed prostaglandin (PGE_2_) and inducible nitric oxide synthase catalyzed NO production on RAW 264.7. The paw edema and body weight was significantly (*P* < 0.001) modulated by the Crocetin in a dose-dependent manner. Crocetin treatment increased the level of red blood cells (RBC), hemoglobin (Hb) and decreased level of white blood cells (WBC), erythrocyte sedimentation rate (ESR), alkaline phosphatase (ALP), serum glutamic pyruvic transaminase (SGPT), and serum glutamic-oxaloacetic transaminase (SGOT) parameters, with reduction of TNF-α, IL-6, and IL-1β.The protective effect of crocetin was substantiated with a reduction in expression of IL-6, IL-1β, VEGF, and TNF-R1, respectively. Crocetin also increased the HO-1/Nrf-2 and decreased the nuclear factor kappa-B (NF-κB) mRNA, protein expression. On the basis of the result, we can conclude that the reduction of HO-1/Nrf-2 expression, as well as inflammatory mediators, may be involved in the protective effect of Crocetin in the CFA model.

## Introduction

Various stimuli such as toxic pathogens, noxious chemical and mechanical agents play a significant role in the inflammatory reaction and create a situation of tissue damage and infection (Eng and Lee, 2003). Macrophages play a significant role in the expansion, resolution, and maintenance of inflammation (Xu et al., 2003). Various stimuli viz., bacterial endotoxin lipopolysaccharides (LPS), interferon gamma (IFN-γ), extracellular matrix proteins, pro-inflammatory (IL-1β, TNF-α, and IL-6) and other chemical mediators (Pahan et al., 1998). During the inflammatory reaction, macrophages become secreted and activate due to release of an excessive amount of inflammatory mediators viz., IL-1β, TNF-α, and IL-6; that have been linked to the numerous inflammatory disease including rheumatoid arthritis, Crohn’s disease, cancer, asthma, Alzheimer’s disease, auto-immune pathologies, and psoriasis to name few (Kmieć, 2001).

Activation of few inflammatory enzymes including inducible nitric oxide synthetase (iNOS), cyclooxygenase (COX-2) and secretion of prostaglandin E_2_ (PGE_2_) from arachidonic acid, and have played a significant role in the expansion of oncogenesis and inflammatory reaction (Gungor et al., 2014). Rahman (2002), suggest that the inhibition of overproduction of inflammatory mediators such as TNF-α, PGE_2_, NO, IL-1β, and IL-6 (Park et al., 2013)with up-regulation of COX-2 and iNOS is an imperative target for the prevention and treatment of inflammation and its related complications (Esser et al., 2014).

Typically arthritis is considered as the common inflammatory disease of joint characterized by being restricted joint movement, pain, and inflammation of synovial membrane (Sellam and Berenbaum, 2010). Rheumatoid arthritis (RA) is one of the common chronic inflammatory conditions which persists over months or years in developed countries and affects the synovial joints, a common cause of deformity and disability (Scott et al., 2010; Walsh and McWilliams, 2012). Joints changes, which probably characterize the autoimmune reaction, consisting of inflammatory reaction, erosion of bone, cartilage, and proliferation of synovial. The epidemiology study of arthritis showed the arthritis ratio between male and female is 3:1 and prevalence is in 1% world population (Scutellari and Orzincolo, 1998; Jimenez-Boj et al., 2005).

Anderson hadclaimed that the enhancement of hind paw edema during the adjuvant-induced infection in rats is paralleled and its due to up-regulation of lysosomal enzymes activities in that particular area (Tekieh et al., 2011). The above-discussed enzymes are involved in the degradation of structural macromolecules in cartilage proteoglycans and connective tissue (Martel-Pelletier et al., 2008; Sindhu et al., 2012). They also induce the destruction of extracellular activity by enhancing the activity of lysosomal enzymes. Further, they destroy the cellular structures and may participate in tissue damage in RA (Kaur et al., 2012). Complete Freund’s adjuvant (CFA) is considered as the most reliable model for estimation of an anti-arthritic drug. CFA containing *Mycobacterium tuberculosis* per milliliter sterile paraffin oil (Bhalekar et al., 2015; Kumar et al., 2015). CFA induced the acute inflammation is linked with mast cell activation, leukocyte infiltration and secretion of free radicals and cytokines in circulation. CFA induced rheumatoid arthritis (RA) characterized by rapid activation of macrophage and secretion of various enzymes into the circulation playing a significant role in the vascular destruction, fibrosis and tissue destruction over a period of time (Harford et al., 2011; Kasimanickam et al., 2014).

Researchers are in the opinion that macrophage-derived cytokines like TNF-α, IL-6, and IL-1β are directly involved in every phase of inflammatory reactions linked with the pathogenesis of RA initiating from autoimmunity in peri-articular phase to upholding joining tissue destruction and chronic inflammation of synovitis (Scott et al., 2010). As on date available treatment of RA is steroidal, non-steroidal and immunosuppressive drugs which are used to control the inflammation symptoms and pain, they are associated with various undesirable side effects. With these obstructions, the researcher focuses on their research on alternative therapies, such as herbal and herbal based drugs. Now, 80% of the World population used the plant-based drug for the effective treatment of pain associated with the RA (Sahoo et al., 2010; Mori et al., 2011; Boissier et al., 2012).

Crocetin (carotenoid) extracted from the saffron flower and its medicinal properties have been tested in Chinese medicine (Bhandari, 2015). Multiple studies have suggested the role of spices in the reduction of chemical inflammation in the rodent. Very rare studies suggest that the saffron also inhibit the expansion of inflammation in the mice (Festuccia et al., 2014; Bhandari, 2015). Glycoprotein considers as the important compound which is involved in the cellular function and play an important role in the surface properties of cells and also involved in the tumorigenesis inflammation and mediator of immunological specificity (Gutheil et al., 2012). The available literature suggests that the carbohydrate moieties of glycoproteins have been implicated in the movement of metabolites inside the cell membrane and also create a direct relationship between the tumorigenesis, inflammation, and glycoprotein (Gutheil et al., 2012; Festuccia et al., 2014; Bhandari, 2015). To our knowledge, this is the first attempt to scrutinize the anti-inflammatory effect of crocetin in LPS induced RAW 264.7 macrophages and CFA induced chronic inflammation.

## Materials and Methods

### Chemicals

Fetal bovine serum (FBS), Dulbecco’s modified eagle’s medium (DMEM), streptomycin and penicillin were obtained from Sigma-Aldrich, United States Crocetin, MTT (4,5-dimethylthiazol-2-yl)-2,5-diphenyltetrazolium bromide), LPS, Indomethacin, and dimethylsulfoxide (DMSO) were purchased from the Sigma-Aldrich, United States. QuantiTect SYBR Green and High capacity cDNA reverse transcription kits were purchased for real-time PCR. Monoclonal antibodies against p-NF-kB p65, NF-kB p65, iNOS, TNF-α, COX-2, and RANKL were procured from the Cell Signaling Technology, Beverly, MA.

### *In vitro* Studies

#### Cell Culture

RAW 264.7 macrophages cells were used for the *in vitro* activity. The cells were diluted to a density of 2 × 10^5^ cells/mL into the 6 flat bottom plates and incubated at 37°C for 24 h in CO_2_ (5%) environment. The cells were treated with the various dilution (12.5, 25, 50, 100, 200, and 400 μg/mL, dissolved in media) of crocetin and incubated for 24 h. After that, the cells were treated with the lipopolysaccharide (LPS) (1 μg/mL) and again incubated for 24 h. The culture medium was collected for pro-inflammatory cytokines (IL-1β, TNF-α, and IL-6), prostaglandin-E_2_ (PGE_2_), nitric oxide (NO) and anti-inflammatory cytokines (IL-10). DMEM medium (cells without the LPS and sample) and 1% μg/mL LPS (without cells) were used as the blank and positive controls, respectively (Heo et al., 2010).

#### Estimation of Prostaglandin-E_2_ (PGE_2_)

Enzyme-linkedimmunosorbent assay (ELISA) was used for the estimation of prostaglandin-E_2_ (Cayman Co., Ann Arbor, MI, United States) concentration in LPS stimulated RAW 264.7 cells. Briefly, the samples and standards drug with dilution 1:100, were added into the 96 well plates, precoated with mouse IgG antibodies, additionally treated with alkaline phosphatase conjugated prostaglandin-E_2_ antibodies and incubated at 37°C on the plate shaker for 3 h (Yoon et al., 2009). After the incubation, the cells were washed with the buffer to remove excess reagent and pNpp (p-nitrophenyl phosphate) substrate (200 μL) followed by addinto each well plate and incubated for 60 min 50 μL was added to stop the reaction and the intensity of the yellow color was estimated immediately at multimode microplate reader at 405 nm and the concentration of PGE_2_was determined via using the PGE_2_ standard curve (Zong et al., 2012).

#### Estimation of Nitric Oxide (NO) Level

Griess diazotization reaction was used for the estimation of the concentration of nitrite by the reported method of Bryan and Grisham (2007), with minor modification. Briefly, cell culture medium (150 μL) was incubated with the equal quantity of Griess reagent (0.1% N-1-(naphthyl) ethylenediamine- dihydrochloride, 1% sulfanilamide in 2.5% phosphoric acid) at 37°C for 30 min and the absorbance was estimated at the 548 nm. Sodium nitrite (NaNO_2_) standard curve was used for the estimation of the concentration of nitrite in treated cells.

#### Estimation of Inflammatory and Anti-inflammatory Cytokines

The inflammatory cytokines (IL-1β, TNF-α, IL-6) and anti-inflammatory cytokines (IL-10) levels were estimated in cell culture supernatant by kit as per the instruction of manufacture (Perpro Tech Inc., Rocky Hill, NJ, United States). Briefly, the samples including 1:50 for IL-6, 1:200 for TNF-α and pure sample for IL-1β and IL-10 were used for the analysis. The standard curves for IL-1β, TNF-α, IL-10, and IL-6 were generated by standard curve plot.

#### Estimation of COX-2 and iNOS

For the estimation of iNOS and COX-2 inhibitory activity, RAW 264.7 cells procured from the American Type Culture Collection, and were harvested in DMEM supplement along with 1% antibiotics (streptomycin and penicillin) 10% FBS under the 5% CO_2_ at 37°C, the cells were activated LPS method with minor modification. Briefly, 96 well plates were used for incubation of cells. After pre-incubation, the test samples and LPS were added to these cells and incubated for the next 24 h. Test compound dissolved in DMSO and diluted with serum-free DMEM for appropriate concentrations and DMSO was used to for adjusting the final concentration 0.1% (v/v). Further, MTT assay was performed for the estimation of cell viability (Chen et al., 2008; Zong et al., 2012).

#### Animals

Swiss albino Wistar rats (100–125 g) were used in our experimental study. The rats were obtained from the central animal house and were kept in the standard condition (single polypropylene cage) (temperature 25 ± 2; relative humidity 60–80 and 12/12 h dark/light cycle). The animals received the standard pellet diet and water *ad libitum*. This study was approved by the Institutional Animal Ethical Committee (CS/2017/278/02).

### Experimental Study

#### CFA Induced Chronic Inflammation Model

The rats were divided into 6 groups and with 10 rats in each group in the following set.

Group I: Normal control treated with saline only

Group II: Normal control received crocetin (20 mg/kg)

Group III: Arthritis control received saline only

Group IV: Arthritis and treated with crocetin (5 mg/kg)

Group V: Arthritis and treated with crocetin (10 mg/kg)

Group VI: Arthritis and treated with crocetin (20 mg/kg)

Group VII: Arthritis and treated with indomethacin (10 mg/kg)

All group rats received the single intradermal injection of CFA (0.5% w/v) and received the oral treatment of crocetin for 28 days except Groups I and II (Kumar et al., 2016). The hind paw joint diameters and body weight were estimated at regular interval. After completion of the protocol, the blood samples were collected and sacrificed for histopathology (Kuroki et al., 2011; Randell and Daneshtalab, 2016).

#### Evaluation of the Arthritis Index

Kumar et al. (2016), a published method was used for the estimation of the arthritis index via minor modification. The arthritis index estimated on the visual scoring system. We use the scale 0–4, scale 0: unchanged, scale 1 erythema of limb and mild swelling, scale 2: erythema of limb and moderate swelling: score 3: erythema of lib and coarse swelling and score 4: the inability of limb and gross deformity. The visual score total more than 1 suggest arthritis and maximum score reach 16.

#### Sample Preparations

For the estimation of different biochemical parameters, the rats were anesthetized at the end of the experimental study and blood samples were collected via puncturing the retro-orbital plexus. The blood samples were centrifuged at 10,000 rpm for 10 min to collect the serum for estimation the different parameters.

#### Hematological Parameters

The collected blood samples further used for the estimation of red blood cells (RBC), white blood cells (WBC), erythrocytes sedimentation rate (ESR), and hemoglobin (Hb), via using the published method (Kaithwas and Majumdar, 2010; Kaithwas et al., 2012; Gautam et al., 2016).

#### Biochemical Parameters

Hepatic parameters such as alkaline phosphatise (ALP), serum glutamic pyruvic transaminase (SGPT), and serum glutamic-oxaloacetic transaminase (SGOT) were estimated via using the available kits (Span Diagnostic Reagent Kit, India) via using the manufacture instruction.

#### Antioxidant Parameters

Antioxidant parameters including glutathione (GSH), catalase (CAT), and superoxide dismutase (SOD) were estimated as per the user manual of standard kits (Sigma-Aldrich, St. Louis, MO, United States).

#### Quantitative Real-Time PCR Determination

Trizol reagent (Sigma-Aldrich, St. Louis, MO, United States) was used for the extraction of total RNA from the paw tissue of CFA induced arthritic rats and UV spectrophotometer was used for the estimation the concentration of Isolated RNA followed by gel electrophoresis was used for the determination of cDNA synthesis via using the RevertAid First Strand cDNA synthesis kit (Thermo Scientific Scientific, Waltham, MA, United States). The primers for real-time PCR were designed from the individual mRNA transcript via using Primer 3 input. Nrf-2 (F-GCCTTCCTCTGCTGCCATTAGT, R-TCGGCTGGGACTTGTGTTCAGT), HO-1 (F-ACCCCACCAAGTTCAAACAGC, R-CCTCTGGCGAAGAAACTCTGTC), GAPDH (F-TGGAAGATGGTGATGGGTTT, R-AGACAGCCGCATCTTCTTGT), and NF-κB (F-TTCTGGTGCATTCTGACCTTGC, R-GAGGAAGGCTGTGAACATGAGG). SYBR Green PCR Master Mix was performed for real-time quantitative PCR via using the manufacture instruction. The fold changes in the gene expression levels of targeted genes were estimated with normalization of GAPDH values and each gene estimation performed in triplicates.

#### Statistical Analysis

Different between the groups were estimated via using the one-way analysis of variance (ANOVA) using the GraphPad Prism 7 software for the window. All the data are presented with the S.E.M and *post hoc* testing was performed for inter-group comparisons using the significant difference. Where *P*-values such as *p* < 0.05, *p* < 0.01, and *p* < 0.001 are considered as significant.

## Results

### Effect of Crocetin on the Cell Viability of RAW 264.7 Macrophages

**FIGURE 1:**
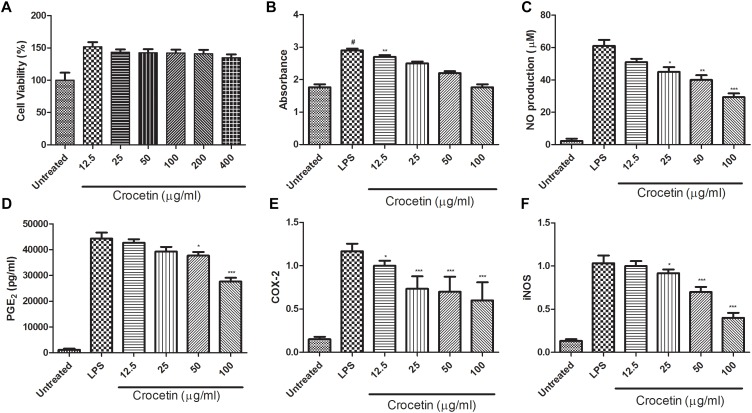
**(A)** Showed the effect of crocetin on viability of RAW 264.7 macrophages. **(B)** Showed the effect of crocetin on phagocytic activity of RAW 264.7 macrophages, **(C)** showed the effect of crocetin on NO production of RAW 264.7 macrophages, **(D)** showed the effect of crocetin on PGE_2_ of RAW 264.7 macrophages, **(E)** showed the effect of crocetin on COX-2 production of RAW 264.7 macrophages and **(F)** showed the effect of crocetin on iNOS production of RAW 264.7 macrophages. The data are presented as mean ± SEM. No significant significance was found in the control and treated groups as scrutinized via one way ANOVA with Dunnett’sposthoc test and designated as ^∗^*p* < 0.05, ^∗∗^*p* < 0.01, ^∗∗∗^*p* < 0.001. ^#^*p* < 0.05 indicates the difference between the control and the LPS-treated group.

Figure 1 are presents the effect of the crocetin on the cytotoxicity of RAW 267.4 macrophages. MTT assay was used to scrutinize the cell viability on RAW 267.4 macrophages. The result from Figure 1 clearly exhibits that the different concentration of crocetin (12.5–400 μg/mL) did not demonstrate any cytotoxic effect on RAW 267.4 cells.

### Effect of Crocetin on Phagocytic Activity of RAW 264.7 Cells

Neutral red uptake assay was used to estimation the phagocytic activity of crocetin on RAW 267.4 cells. Figure 1B revealed that the crocetin treated cells significantly (*P* < 0.001) boosted the phagocytic activity on RAW 267.4 cells as a compared to the untreated cells. Figure 1B clearly showed that the lower dose of crocetin (12.5 μg/mL) significantly (*P* < 0.001) excited the phagocytosis, on the other hand, higher dose of crocetin did not show much effect on the phagocytosis in RAW 267.4 cells.

### Effect of Crocetin on NO Production

Figure 1C demonstrated the effect of crocetin on the NO production on LPS induced RAW 267.4 cells. Figure 1C showed that the minimum amount of NO was release when macrophages were alone cultured and LPS stimulated the macrophages NO production (61 ± 5.46 μM). On the other hand, crocetin (100 μg/mL) significantly (*P* < 0.001) reduced the production of NO (29.73 ± 4.56 μM) as a compared to LPS control. Crocetin (12.5 μg/mL) alter the production of NO but the data was not significant.

### Effect of Crocetin on PGE_2_ Production

Figure 1D revealed that the LPS induced macrophages increased the production of PGE_2_ up to (44,374 ± 1244 pg/mL) when compared to untreated cells and crocetin significantly (*P* < 0.001) down-regulated the PGE_2_ production in LPS induced macrophages at a dose of 100 μg/mL.

### Effect of Crocetin on COX-2 and iNOS

During the inflammation, the level of COX-2 and iNOS was boosted and increased inflammatory reaction was observed. The same momentum was observed in our experimental study. LPS induced macrophages group showed the increased level of COX-2 and iNOS as compared to untreated cells and dose-dependent treatment of crocetin significantly (*P* < 0.001) down-regulated the COX-2 level as compared to LPS induced macrophages group (Figure 1E).

Similar results were observed in the iNOS concentration. LPS induced group macrophages exhibited the increased concentration and by dose-dependent treatment of crocetin reduced the iNOS concentration in LPS induced macrophages (Figure 1F).

### Effect of Crocetin on Pro-inflammatory Cytokines

**FIGURE 2:**
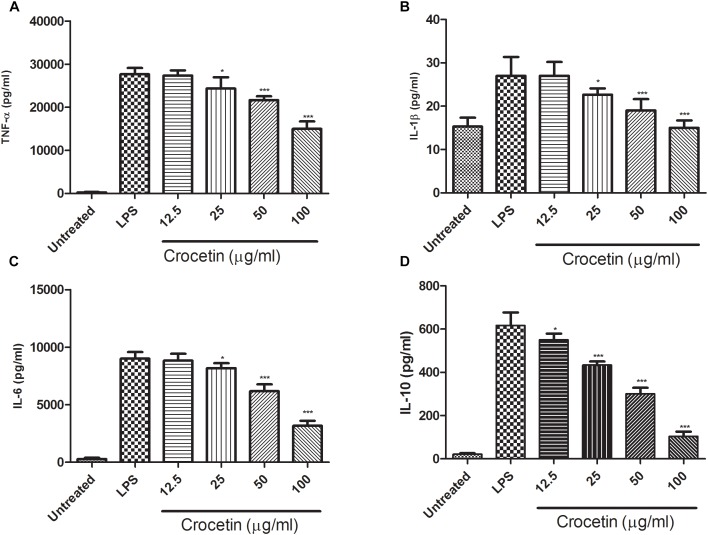
Showed the effect of crocetin on proinflammatory and inflammatory mediators of RAW 264.7 macrophages. **(A)** TNF-α, **(B)** IL-1β, **(C)** IL-6, and **(D)** IL-10. The data are presented as mean ± SEM. No significant significance was found in the control and treated groups as scrutinized via one way ANOVA with Dunnett’s *post hoc* test and designated as ^∗^*p* < 0.05, ^∗∗^*p* < 0.01, ^∗∗∗^*p* < 0.001.

During the inflammation, a lot of pro-inflammatory mediator such as (IL-1β, IL-6, and TNF-α) and inflammatory mediator IL-10 are boosted and start the expansion of inflammation reaction. The same result was observed in our experimental study. Figure 2 exhibited the increased level of IL-1β (27 ± 2.85 pg/mL), IL-6 (9004 ± 753 pg/mL), TNF-α (27,695 ± 1203 pg/mL)and IL-10 (616.75 ± 11.56 pg/mL) and crocetin (100 μg/mL) significantly (*P* < 0.001) down-regulated the level of IL-1β (14.9 ± 2.67 pg/mL), IL-6 (3187 ± 578 pg/mL) and TNF-α (15,004 ± 936 pg/mL) and IL-10 (104.6 ± 7.64 pg/mL) at end of the experimental study (Figure 2).

### Effect on the Body Weight

Table 1 showed the effect on the body weight of all group rats. CFA induced group rats showed the reduced body weight at end of the experimental study and dose-dependent treatment of Crocetin showed significantly (*P* < 0.001) increased body weight at end of the experimental study. Indomethacin-induced group rats showed the increased body weight at end of the experimental study.

**Table 1:** showed the effect of crocetin and indomethacin on body weight of normal and CFA induced arthritic rats.

S. No	Groups	Time (Days)				
		Day 0	Day 7	Day 14	Day 21	Day 28
1	NC	100 ± 4.35	109.34 ± 5.43	119.23 ± 6.54	130.83 ± 7.94	141.34 ± 9.34
2	NC+Crocetin (10 mg/kg)	103.2 ± 5.43	113.45 ± 6.94	125.3 ± 5.84	136.34 ± 6.78	147.38 ± 83
3	CFA	104.4 ± 5.67	101.3 ± 6.54	98.6 ± 5.67	96.4 ± 6.47	94.54 ± 5.67
4	CFA+Crocetin (2.5 mg/kg)	105.54 ± 6.76ns	104.37 ± 5.43*	102.45 ± 5.93*	102.67 ± 6.54**	104.56 ± 5.67***
5	CFA+Crocetin (5 mg/kg)	103.83 ± 5.83ns	102.53 ± 7.34*	105.45 ± 6.54**	106.52 ± 5.67***	109.34 ± 6.54***
6	CFA+Crocetin (10 mg/kg)	103.45 ± 5.67ns	104.34 ± 5.67*	111 ± 5.78***	117.34 ± 4.56***	125.67 ± 5.63***
7	CFA+Indomethacin (10 mg/kg)	103.92 ± 4.78ns	104.67 ± 5.63*	112.34 ± 5.78***	119.3 ± 5.43***	126.43 ± 6.56***


*All values are presented as mean ± SEM. Statistical analysis by one-way ANOVA followed by post hoc multiple comparisons.*^∗^*p < 0.05*, ^∗∗^*p < 0.01, and*^∗∗∗^*p < 0.001.*

### Effect on Paw Edema

CFA induced group rats showed the increased paw edema at end of the experimental study (Table 2). The paw edema increased after the 7 days induction of CFA induced arthritis, which further increased till 25 days and remain unchanged at end of the experimental study. Crocetin treatment showed the reduction in the paw edema after 14 days and also reduced the paw edema at end of the experimental study. Indomethacin-induced group rats showed the reduction of the paw edema at end of the experimental study.

**Table 2:** showed the effect of crocetin and indomethacin on joint diameter of CFA induced arthritic rats.

S. No	Groups	Joint diameter (mm)			
		Day 7	Day 14	DAY 21	Day 28
1	CFA	1.26 ± 0.11	1.16 ± 0.15	1.13 ± 0.16	1.03 ± 0.12
2	CFA+Crocetin (2.5 mg/kg)	1.23 ± 0.1ns	1.04 ± 0.14***	0.90 ± 0.11***	0.60 ± 0.09***
3	CFA+Crocetin (5 mg/kg)	1.23 ± 0.13ns	0.96 ± 0.11***	0.80 ± 0.9***	0.5 ± 0.7***
4	CFA+Crocetin (10 mg/kg)	1.16 ± 0.12ns	0.8 ± 0.09***	0.55 ± 0.06***	0.26 ± 0.02***
5	CFA+Indomethacin (10 mg/kg)	1.1 ± 0.11ns	0.7 ± 0.05***	0.5 ± 0.03***	0.2 ± 0.01***


*All values are presented as mean ± SEM. Statistical analysis by one-way ANOVA followed by post hoc multiple comparisons. ^∗^p < 0.05, ^∗∗^p < 0.01, and ^∗∗∗^p < 0.001. NC and NC rats treated with crocetin (10 mg/kg) did not show any sign of increase joint diameter.*

### Effect on Arthritis Score

**FIGURE 3:**
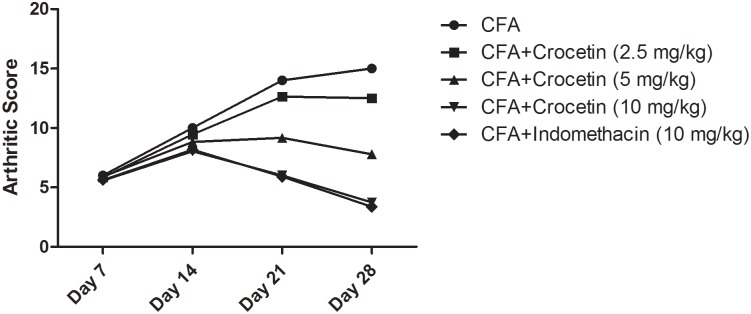
Showed the arthritic score of crocetin and indomethacin on the normal and CFA induced arthritic rats. All values are presented as mean ± SEM. Statistical analysis by one-way ANOVA followed by *post hoc* multiple comparisons.

Figure 3 exhibited the arthritis score of all group of rats except normal control and normal control treated with crocetin (10 mg/kg). Normal and normal control treated with crocetin (10 mg/kg) did not show any sign and symptom of arthritis. CFA induced group rats showed the arthritis score 15.3 at day 28 and confirm arthritis in this group rats. Dose-dependent treatment of crocetin significantly (*P* < 0.001) reduced the arthritis score of 12.6, 7.9, and 3.4 at a dose of 2.5, 5, and 10 mg/kg. A similar result was observed in the indomethacin-treated group of rats.

### Effect on Antioxidant Parameters

**FIGURE 4:**
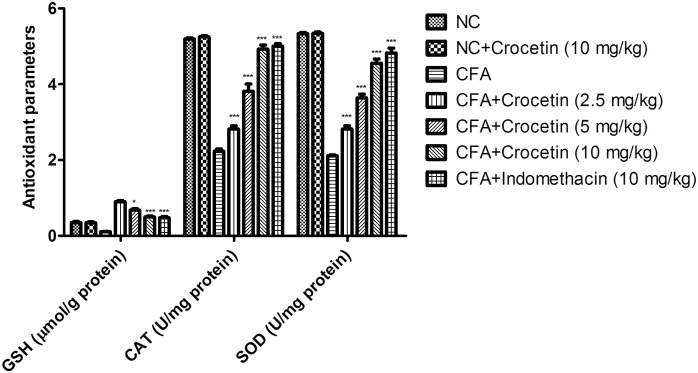
Showed the effect of crocetin and indomethacin on the level of GSH, CAT and SOD on the normal and CFA induced arthritic rats. All values are presented as mean ± SEM. Statistical analysis by one-way ANOVA followed by *post hoc* multiple comparisons. ^∗^*p* < 0.05, ^∗∗^*p* < 0.01, and ^∗∗∗^*p* < 0.001.

Figure 4 exhibits the antioxidant level of normal and CFA treated group rats. CFA induced group rats showed the reduced level of SOD, CAT, and GSH and dose-dependent treatment of crocetin significantly (*P* < 0.001) increased the level of SOD, CAT, and GSH at end of the experimental study.

### Effect on Biochemical Parameters

**FIGURE 5:**
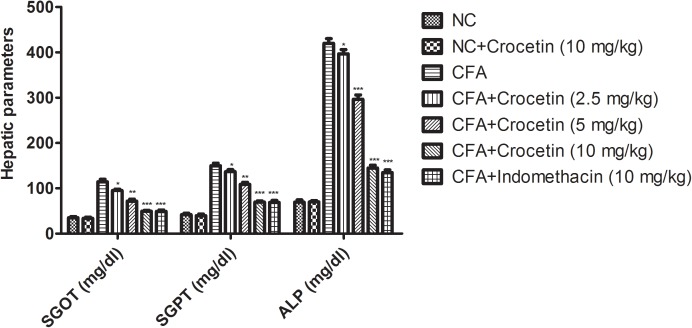
Showed the effect of crocetin and indomethacin on the level of SGOT, ALP, and SGPT on the normal and CFA induced arthritic rats. All values are presented as mean ± SEM. Statistical analysis by one-way ANOVA followed by *post hoc* multiple comparisons. ^∗^*p* < 0.05, ^∗∗^*p* < 0.01, and ^∗∗∗^*p* < 0.001.

Figure 5 showed the level of ALT, AST, and ALP were significantly (*P* < 0.001) increased and the total protein level was significantly (*P* < 0.001) decreased in the CFA induced arthritis group rats. Crocetin treatment showed the significantly (*P* < 0.001) alteration of biochemical parameters in a dose-dependently manner. The same result was observed in the indomethacin-treated group rats.

### Effect on Hematological Parameters

**FIGURE 6:**
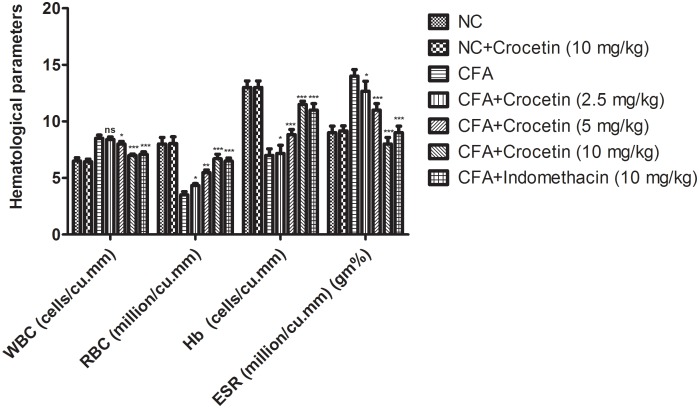
showed the effect of crocetin and indomethacin on the level of RBC, WBC, Hb and ESR on the normal and CFA induced arthritic rats. All values are presented as mean ± SEM. Statistical analysis by one-way ANOVA followed by *post hoc* multiple comparisons. ^∗^*p* < 0.05, ^∗∗^*p* < 0.01, and ^∗∗∗^*p* < 0.001.

Figure 6 showed the effect of Crocetin and indomethacinon the hematological parameters. CFA induced showed the reduced the level of RBC, Hb and enhanced level of WBC, ESR and in dose-dependent manner treatment of Crocetin significantly (*P* < 0.001) reduced the level of WBC, ESR and increased the level of RBC, Hb at end of the experimental study. A similar trend was observed in the indomethacin-induced group rats.

### Gene Expression Estimation via Real-Time PCR

**FIGURE 7:**
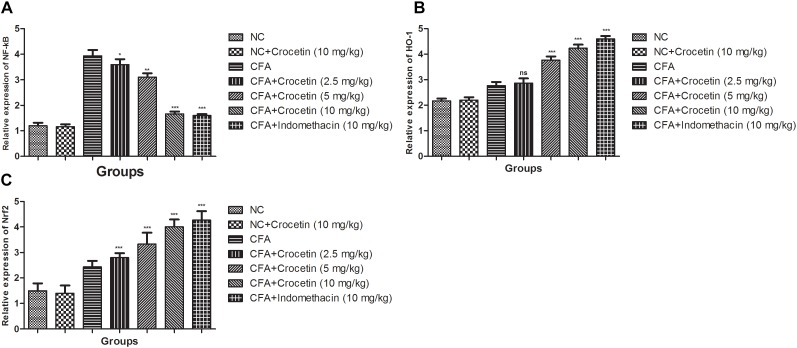
Effect of crocetin and indomethacin on mRNA expression of **(A)** NF-κB, **(B)** HO-1, and **(C)** Nrf-2 in normal and CFA induced rats. All values are presented as mean ± SEM. Statistical analysis by one-way ANOVA followed by *post hoc* multiple comparisons. ^∗^*p* < 0.05, ^∗∗^*p* < 0.01, and ^∗∗∗^*p* < 0.001.

Figure 7 showed the mRNA expression levels of HO-1, Nrf-2, and NF-κB of crocetin and indometacin treated group rats. A marked increased level of NF-κB and reduced level of HO-1, Nrf-2 was observed in the CFA induced group rats and crocetin and indomethacin treatment showed the alteration in the gene expression of HO-1, Nrf-2, and NF-κB, respectively.

## Discussion

This study was an effort to scrutinize the anti-inflammatory effect of crocetin in *in vitro* and *in vivo* experimental model by LPS stimulated RAW 267.4 macrophages and CFA induced chronic arthritis model, respectively. Crocetin exhibited the inhibitory potential against the COX-2, iNOS, pro-inflammatory (IL-1β, IL-6, IL-10, and TNF-α) in *in vitro* assays, a potent *in vivo* potential in a rat model, suggested the protective and therapeutic effect on inflammatory tissue. Lipopolysaccharide (prototypical endotoxin) isolated from the Gram-negative bacterial membrane can directly activate the endothelial cells, macrophages and complement triggering generation of inflammatory mediators viz., COX-2, iNOS, pro-inflammatory (IL-1β, IL-6, IL-10, and TNF-α), respectively.

Excessive generation of NO, play a significant role in the provocation of chronic inflammatory and circulatory shock diseases, like inflammatory lung disease, septic shock, colitis and inflammatory hepatic dysfunctions (Zhang et al., 2017). Many of these disease conditions show the quick onset and expansion, frequently that result in the failure of conventional anti-inflammatory treatment with high mortality rates, a concurrent down-regulation of NO production pathways (Zhang et al., 2012), as exposed by crocetin may suggest managing the quick progression of inflammatory reactions. *In vitro* macrophages cell model or other cell lines model are very useful to study the high-level production of NO (Zhang et al., 2012). On the basis of the result, we can conclude that crocetin inhibits the LPS stimulate NO production via suppression of inflammatory expression including COX-2 and iNOS (Figure 1). On the other hand, it was also scrutinized the cytotoxic effect of crocetin in RAW 267.4 macrophages by MTT assay that suggest that its minimum dose did not influence the viability of RAW 267.4 cells. Therefore, the suppression of LPS induced NO production via crocetin was not the result of a probable cytotoxic upshot on these cells.

For confirmation of NO and PGE_2_ inhibitory mechanism of Crocetin, it showed the significant inhibition of PGE_2_ and slight reduction of NO production. The current finding suggests that Crocetin directly down-regulated the COX-2 and also affected the iNOS production in a different way (Hseu et al., 2005; Yoon et al., 2009; Heo et al., 2012).

Various researchers suggest that the excessive generation of PGE_2_ and NO play a significant role in the provocation of chronic disease including pulmonary and hepatic dysfunction disease (Ohgami et al., 2003). Secretion of PGE_2_ and NO play an important role in the alteration of molecular mechanisms that regulate the PGE_2_ generation pathway (Heo et al., 2012). The available reports suggest that the generation of both PGE_2_ and NO was blocked via NOS inhibitors in RAW 267.4 macrophages cells, this suppressive effect was reverted via co-incubation with NO synthesis precursor (L-arginine) (Zhang et al., 2012). Therefore, reduction of iNOS via non-selective NOS inhibitors calmed the generation of PGE_2_ and NO simultaneously in LPS induced macrophages, suggesting the secretion of endogenous NO from the macrophages that exerted a stimulatory action on boosting the PGE_2_production (Hseu et al., 2005; Yoon et al., 2009; Heo et al., 2012). On the contrary, activation of COX, turn into the alteration of the L-arginine-NO production pathway, whereas the suppression of COX down-regulate the NOS effect in human platelets. Our results are the outcome of cross-talk between PGE_2_ and NO pathways.

CFA induced RA model used for the estimation of pharmacology and pathophysiological control of inflammatory processes, as well as the, scrutinize the anti-arthritic effect of drugs. CFA induced RA showed the chronic inflammation, in the first 2–4 days induced the local inflammation and after that induced the chronic inflammation which remains for next few weeks (Bernardi et al., 2009; Rayhana et al., 2014). CFA has been shown the number of clinical, immunological and chronic feature similar to human arthritis. The primary reaction of the CFA showed the expansion of inflammation and developed the arthritic nodules in the tail and ear and persist the swelling in contralateral and showed the delayed response (secondary reaction). CFA induced RA rats showed the enhancement of paw joint in from day 3 and remain increase until 28 days. CFA control group rats showed the increase joint diameter till 28 days and suggest the delayed immunological flare in the RA disease (Kuroki et al., 2011; Wang et al., 2012).

Currently, available treatment for the arthritis is NSAIDS (Non-Steroidal Anti-Inflammatory Drugs) viz., indomethacin, aceclofenac, phenylbutazone, ibuprofen etc., DMARDs (Disease Modifying Anti-Rheumatic Drugs) viz., cyclosporin A, methotrexate etc, Anticytokine therapy such as infliximab, adalimumab etc and immune suppressive drugs are commonly used to control the inflammation and pain during arthritis (Kumar et al., 2016). Indomethacin is the first choice of the drug due to its specific action on COX and PGE_2_. But this drug has a limitation due to gastro intestinal irritation, diarrhea, rectal irritation, skin rash, hematologic toxicity and loss of response with chronic use (Kaithwas and Majumdar, 2010). It is moreover desirable that the more potent antiarthritic drugs be investigated for specific action on the inflammation and pain during arthritis.

During the arthritic condition, hematological parameters altered due to the expansion of disease. A similar result was observed in the CFA induced group rats. CFA group rats exhibited the increased level of ESR, WBC and reduced level of Hb RBC (Kumar et al., 2016). During the arthritic condition, the level of RBC and Hb decrease and create the anemia condition to the patient due to the deformability of erythrocyte (shorten the lifespan of erythrocyte). During the disease, the level of Hb was decreased due to the expansion of disease, resultant start the destruction of premature RBCs and reduction of erythropoietin (reduced the level of bone marrow erythropoietin) (Kumar et al., 2017). A similar condition was observed in our study. Another parameter such as WBC, its considers as the significant marker of the immune system which is linked with the induction of inflammation and its related other diseases. During the arthritic disease condition, IL-1β mediated WBC level increase and start the generation of colony stimulating factor, granulocyte and inflammatory macrophages (Jeyadevi et al., 2013). Crocetin significantly (*P* < 0.001) reduced the granulocytes and migration of inflammation macrophages at dose-dependent manner. Increased level of ESR start the generation of endogenous proteins and actively take part in the progression of the disease. Result suggests that the dose-dependent treatment of crocetin altered the hematological condition near to the normal level and suggest the anti-arthritic effect.

Various researchers suggest that the hepatic parameters such as ALP, SGPT, and SGOT are the tool for the estimation of the anti-arthritic effect of the tested drug (Kumar et al., 2016). Serum SGPT and SGOT level played an important role in the formation of biologically active chemical mediators such as bradykinins in the inflammatory process and suggest a positive relation between the enhanced activity of serum ALP and RA activity (Kaithwas and Majumdar, 2010). The aminotransferases enzymes were significantly boosted in disease rats since there is the marker of kidney and liver impairment, which are also considered as the features of adjuvant arthritis (Gautam et al., 2016). During arthritis, the level of ALP increased in the bone fraction and liver or may be due to increasing the level of both isoenzymes. This in turn implicates a localized bone loss in the form of bone erosion and periarticularosteopenia, as the enzyme is released into circulation in the course of bone formation and resorption (Kaithwas and Majumdar, 2010). Concentration-dependent treatment of crocetin significantly decreased the activity of hepatic enzymes such as SGOT, SGPT, ALP and suggest the reduced the bone loss and provide the protection to the organ against adjuvant-induced arthritis in the rats. From the result, we can say that crocetin provided the anti-arthritic effect against the CFA induced arthritic rats due to alteration of biochemical parameters.

In the current experimental study, we scrutinized the macrophage-derived pro-inflammatory cytokines such as IL-6, TNF-α, and IL-1β. Various researchers suggested that the level of pro-inflammatory cytokines increase during the CFA induced RA (Qureshi et al., 2011). These cytokines boosted the local systemic inflammatory response and induced the cartilage, infiltration of immune cells and start the degradation of joint via degrading the enzymes such as metalloproteinases (MMPs) (Impellizzeri et al., 2012). For confirmation of the exact mechanism of action, in the current experimental study, we evaluated the pro-inflammatory cytokines in synovium and serum (Hübner et al., 1996; Fernandes et al., 2002; Kim et al., 2007; Neurath, 2014). The level of TNF- α was up-regulated in the arthritis patient. The up-regulated level of TNF- α start the damage of gastrointestinal at indomethacin group rats. CFA induced group rats showed the up-regulation of TNF- α and dose-dependent treatment of crocetin showed the down-regulation of pro-inflammatory cytokines level.

CFA induced control group rats showed that the rat’s hind paw increased in diameter till the end of the experimental study. The increase in joint diameters increase is a result of arthritis disease (Hussein et al., 2012; Kumar et al., 2017). CFA induced group rats showed the reduced body weight and crocetin treatment showed the increased body weight and suggests the anti-arthritic effect. CFA induced showed the decreased body weight due to deficient absorption of nutrient via intestine and the treatment showed the increased body weight suggest the normalizing process of the intestine (Jalalpure et al., 2011; Nair et al., 2011; Suke et al., 2013; Jiang et al., 2014). The possible mechanism may be improved the level of nutrient from intestine absorption and down-regulation of distress induced through the severity of arthritis.

In the current investigation, the joint inflammation of crocetin treated group rats decreases at the end of the experimental study. CFA control rats showed the increased level of TNF- α and dose-dependent treatment of crocetin exhibited the reduction of TNF-α, however, there was a higher TNF- α level was observed in the indomethacin group (Gautam et al., 2016). The increased level of TNF- α found in the indomethacin group due to gastrointestinal damage, which further caused the gastrointestinal ulcer. Inflammatory mediators such as IL-6, IL-1β, VEGF, and TNF-R1 are correlated with our finding in the animal investigation, as the expression of IL-6, IL-1β, VEGF, and TNF-R1 were observed to be attenuated in the synovial membrane of crocetin and indomethacin group (De Groote et al., 1992; Stevens et al., 2008; Nadeau et al., 2011; Studer et al., 2011). Indomethacin was more effectual in reducing the joint inflammation via inhibiting the inflammatory mediators. The same results were observed in the crocetin group rats, which showed the reduction of joint inflammation and pro-inflammatory cytokines in a dose-dependent manner. The result suggests that crocetin preventing the destruction of joint and synovial membrane via the different/additional mechanism.

Various researchers suggest that pathogenesis of RA having various inflammatory reactions such as transcription factor, NF-κB, which play a significant role in the joint degradation and inflammation via increased the expression of pro0inflammatory genes viz., IL-6, IL-1β, TNF-α, MMPs, and chemokines (Bonizzi and Karin, 2004; Shakibaei et al., 2007; Choi and Lee, 2010; Ali et al., 2011). The increased level of NF-κB in the synovial joint of animal and human during the RA is well observed. Now, the researcher focuses their research on the RA via inhibiting the NF-κB expression for the treatment of it. In the current investigation, we scrutinized the effect of NF-κB in the control and treated group rats (Makó et al., 2010; Mori et al., 2011). Dose-dependent treatment of crocetin significantly (*P* < 0.001) decreased the expression of NF-κB as compared to CFA induced rats. The current finding suggests that crocetin could be responsible for anti-arthritic activity via inhibition of NF-κB expression.

Nrf2 is considered as the significant transcription factor which was found to numerous set of enzymes including GPx, glutathione S-transferase and HO-1 via binding of DNA sequence called antioxidant response element (ARE), which has the ability to reduced the pro-inflammatory pathway and antioxidant response (Itoh et al., 1999; Li et al., 2004; Jung and Kwak, 2010; Vomhof-DeKrey and Picklo, 2012). Various researchers suggest that cross relationship between NF-κB/IL-1β and Nrf2 and serve the joint destruction and oxidative injury in animals (Lee and Surh, 2005; Vomhof-DeKrey and Picklo, 2012). In the current experimental study, we have found that the crocetin treatment increased the mRNA expression of HO-1 and Nrf2 in CFA induced rats as compared to CFA control group rats and suggesting the suppression of oxidative stress and pro-inflammatory cytokines via altering the oxidative stress and pro-inflammatory cytokines level.

## Conclusion

On the basis of the result, we can conclude that crocetin was an effective agent in attenuating the adjuvant-induced arthritis rats in a concentration-dependent manner, and therefore it could be scrutinized as a probable treatment for human chronic arthritis condition. The results suggest that crocetin is beneficial in the treatment of painful inflammatory and chronic arthritic condition. Additionally, particular identify the gene responsible for the anti-arthritic effect.

## Author Contributions

YL and RK performed the experimental study. JW, YL, and RK interpreted the biochemical data. All the authors equally contributed for the proofreading.

## Conflict of Interest Statement

The authors declare that the research was conducted in the absence of any commercial or financial relationships that could be construed as a potential conflict of interest.

## Abbreviations

ALP: Alkaline phosphatise
CAT: Catalase
CFA: Complete freund’s adjuvant
COX-2: Cyclooxygenase
DMEM: Dulbecco’s modified eagle’s medium
DMSO: Dimethylsulfoxide
ESR: Erythrocytes sedimentation rate
FBS: Fetal bovine serum
GSH: Glutathione
Hb: hemoglobin
HO-1: Heme oxygenase-1
IFN- γ: Interferon gamma
IL-1β: Interleukin-1β
IL-6: Interleukin-6
IL-10: Interleukin-10
iNOS: Nitric oxide synthetase
LPS: Lipopolysaccharides
MMPs: Metalloproteinases
MTT: 4,5-dimethylthiazol-2-yl)-2,5-diphenyltetrazolium bromide
NF-kB: Nuclear factor kappa-B
NO, NaNO_2_: Sodium nitrite; Nitric oxide
Nrf-2: Nuclear factor erythroid 2–related factor 2
PGE_2_: Prostaglandin
pNpp: p-nitrophenyl phosphate
RA: Rheumatoid arthritis
RBC: Red blood cells
SGOT: Serum glutamic-oxaloacetictransaminase
SGPT: Serum glutamicpyruvictransaminase
SOD: Superoxidase dismutase
TNF-α: Tumornecorsis factor-α
TNF-R1: Tumor necrosis factor receptor 1
VEGF: Vascular endothelial growth factor
WBC: White blood cells
